# Towards estimation of CO_2_ adsorption on highly porous MOF-based adsorbents using gaussian process regression approach

**DOI:** 10.1038/s41598-021-95246-6

**Published:** 2021-08-03

**Authors:** Majedeh Gheytanzadeh, Alireza Baghban, Sajjad Habibzadeh, Amin Esmaeili, Otman Abida, Ahmad Mohaddespour, Muhammad Tajammal Munir

**Affiliations:** 1 Surface Reaction and Advanced Energy Materials Laboratory, Chemical Engineering Department, Amirkabir University of Technology (Tehran Polytechnic), Tehran, Iran; 2 Chemical Engineering Department, Amirkabir University of Technology (Tehran Polytechnic), Mahshahr Campus, Mahshahr, Iran; 3 Department of Chemical Engineering, McGill University, 3610 University Street, Montreal, QC H3A 0C5 Canada; 4 Department of Chemical Engineering, School of Engineering Technology and Industrial Trades, College of the North Atlantic—Qatar, Doha, Qatar; 5 College of Engineering and Technology, American University of the Middle East, Egaila, Kuwait

**Keywords:** Climate sciences, Environmental sciences, Chemistry, Engineering, Materials science, Mathematics and computing, Nanoscience and technology

## Abstract

In recent years, new developments in controlling greenhouse gas emissions have been implemented to address the global climate conservation concern. Indeed, the earth's average temperature is being increased mainly due to burning fossil fuels, explicitly releasing high amounts of CO_2_ into the atmosphere. Therefore, effective capture techniques are needed to reduce the concentration of CO_2_. In this regard, metal organic frameworks (MOFs) have been known as the promising materials for CO_2_ adsorption. Hence, study on the impact of the adsorption conditions along with the MOFs structural properties on their ability in the CO_2_ adsorption will open new doors for their further application in CO_2_ separation technologies as well. However, the high cost of the corresponding experimental study together with the instrument's error, render the use of computational methods quite beneficial. Therefore, the present study proposes a Gaussian process regression model with four kernel functions to estimate the CO_2_ adsorption in terms of pressure, temperature, pore volume, and surface area of MOFs. In doing so, 506 CO_2_ uptake values in the literature have been collected and assessed. The proposed GPR models performed very well in which the exponential kernel function, was shown as the best predictive tool with R^2^ value of 1. Also, the sensitivity analysis was employed to investigate the effectiveness of input variables on the CO_2_ adsorption, through which it was determined that pressure is the most determining parameter. As the main result, the accurate estimate of CO_2_ adsorption by different MOFs is obtained by briefly employing the artificial intelligence concept tools.

## Introduction

The concentration of atmospheric CO_2_ has increased from 270 ppm before the industrial revolution to more than 400 ppm today, mainly due to the increasing consumption of fossil fuels^1^. In addition, it is widely believed that CO_2_ has a major role in global climate change^2^. Thus, carbon capture technology has been employed as a promising route to reduce the CO_2_ concentration into the atmosphere and inhibit global warming^3,4^. Several approaches have been studied for CO_2_ capture: membranes^5,6^, chemical absorption^7,8^, physical adsorption^9^, and fluidized bed technologies^10^. However, these methods suffer from some drawbacks, such as high energy consumption, complex regeneration processes, and low CO_2_ capture capacity. In order to build up a long-lasting chance in CO_2_ elimination, an appropriate adsorption medium should provide the following conditions: (1) a periodical structure for both the capture and release of CO_2_ reversibly, (2) high CO_2_ selectivity, (3) optimized CO_2_ adsorption capacities through modifying by chemical functionalization, and (4) thermal, chemical, and mechanical stabilities^11,12^. Metal–organic frameworks (MOFs) have been one of the most applicable porous compounds due to their regulating chemical structure, adjustable chemical functionality, and high thermal stability, allowing potential applications in gas adsorption^13–16^.

MOFs are formed by a combination of two main parts of metal ions or clusters and organic ligands, creating a 3D structure with a network of channels and uniform pores. In addition to the robust 3D structure, the main characteristics of the MOFs are their permanent porosity and modular nature. These features of MOFs support them in adsorbing other molecules as a guest and sustaining their structures with negligible damage^17,18^. In comparison to the other porous materials, the most important advantage of the MOFs is their possibility to design the functionality and the pore size by choosing the metal ion, the functional group, the organic ligand, and the activation method^19^. The properties of MOFs depend on the metal of interest and the linker. For example, MOF-5 or IRMOF-1, containing zinc atoms linked to terephthalic acid molecules, possess a big void for gas capture, while M-dobdc or M-MOF-74 (M = Mg, Ni, Co, Zn), with unsaturated metal centers in their 3D structures, provide extra sites to bond with guest molecules^20,21^. Besides, the pore sizes of MOFs change from several angstroms to a few nanometers based on the diverse organic linkers^15^.

Several studies reported high CO_2_ adsorption capacity for MOF materials, ranging from 8.0 to 10.2 mol/kg at 298 K and 15 bar. CuBTC or HKUST-1 is one of the most explored MOFs for gas adsorption and storage^17,22–24^. To compare the adsorption capacity in zeolites and MOFs, at higher pressures, the adsorption capacity of the benchmark zeolite 13X is much lesser than that of MOFs^22^. Additionally, when the micropore diffusion is the rate control mechanism for CO_2_ adsorption, the adsorption process in NaX and 5A zeolites proceeds slower than in MOF materials^25^. MOFs are promising candidates for gas adsorption applications among the various porous materials based on the mentioned features.

Despite numerous studies reported about gas–solid adsorption systems, investigating this phenomenon from a cohesive viewpoint is still challenging^26^. The experimental studies are time-consuming and costly, through which the instruments' errors affect the adsorption results. On the other hand, many adsorption isotherms are usable just for a specific range of data because they have been developed under simplified conditions assumptions^27^. Accordingly, a comprehensive and accurate model for examining the adsorption of a gas on MOFs should be developed. Intelligent methods (machine learning algorithms), namely, least-square support vector machine (LS-SVM), artificial neural network (ANN), random forest (RF) adaptive neuro-fuzzy inference system (ANFIS), and radial basis function network (RBF), can be possibly hired as an alternative to mathematical models for solving problems precisely and without the experimental works' troubles^28,29^. Compared to the conventional mathematic approaches, the smart models have gained excellent success in solving complex and non-linear optimization problems^30–39^.

In the current study, an intelligent model is used to predict the non-linear system of CO_2_ capture by MOFs materials. For the first time, a machine learning algorithm of GPR with four various kernel functions was developed to evaluate the CO_2_ uptake on MOFs. Thirteen MOFs with different porosity and structural features including: Cu_3_(BTC)_2_, MOF-505, MOF-74, IRMOF-11, beryllium benzene tribenzoate (Be-BTB), MOF-177, IRMOF-1, IRMOFs-3, IRMOFs-6, MOF-2, Cu-BTTri (BTTri^3−^ = 1,3,5-benzenetristriazolate), Mg^2−^ (dobdc) (dobdc^4−^ = 1,4-dioxido-2,5-benzenedicarboxylate), and Co(BDP) (BDP^2−^ = 1,4-benzenedipyrazolate) based on experimental data were studied^17,40^. Pressure, temperature, pore volume, and surface area of MOFs are considered the model's inputs. Several statistical analyses were applied to investigate the established model, while analysis of sensitivity was used to determine the effective factors on the CO_2_ adsorption by MOFs. Additionally, to assess the precision of the proposed GPR models, the predicted results were compared with the experimental CO_2_ adsorption values in the literature.

## Methodology

### Gaussian process regression

This study used the machine learning technique, GPRs model, because they are able to deal with uncertainty in a probabilistic framework (Bayesian) and overcome the complex issues straightforwardly^41,42^. The non-linear GPR models need less training data and can combine new evidence when the available data increases. Typically, the low number of hyper-parameters to optimize through training makes this model less affected by the “overfitting” problem^43^. In the GPR technique, the training sample information determines the parameters of the model. Then, the GPR model is developed via adding the previous information to the modeling procedure and merging the actual (laboratory-measured) data^41^. In contrast to the traditional learning models, the GPR works through computing posterior distributions over models instead of finding the most acceptable match to the experimental data^44^.

Generally, the GPR model is established in this way: if the input and the target variables are represented by x and y, assume $T={\left\{{x}_{T\cdot i}\cdot {y}_{T\cdot i}\right\}}_{i=1}^{n}$ and $L={\left\{{x}_{L\cdot i}\cdot {y}_{L\cdot i}\right\}}_{i=1}^{n}$ as the arbitrarily chosen test and training data sets, respectively. The starting step in the GPR modeling is the following general equation:

1 $$ y_{L \cdot i} = f\left( {x_{L \cdot i} } \right) + \varepsilon_{L \cdot i} \quad i = 1,2,3, \ldots ,n $$

where x_L_ indicates the independent variables and y_L_ represents the targets of the learning data points. The $\varepsilon \sim N(0\cdot {\sigma }_{noise}^{2}{I}_{n})$, σ^2^_noise_ , and I_n_ are the observation noise, the variance of the noise, and the unit array, respectively. Therefore, each measured y is connected to the function f(x) by Gaussian noise model^45^. GPR assumes *f* as a random function that can be entirely defined by its covariance and mean functions. Likewise, we can write:

2 $$ y_{T \cdot i} = f\left( {x_{T \cdot i} } \right) + \varepsilon_{T \cdot i} \quad i = 1,2,3, \ldots ,n $$

where x_T_ denotes the independent variables, and y_T_ is the targets of the testing data sets. Also, the f(x) is distributed as a Gaussian process with covariance function k(x, x′) (also called kernel function) and mean function m(x) ^45^:

3 $$ f\left( {x_{L \cdot i} } \right) \sim GP\left( {m\left( x \right) \cdot k\left( {x \cdot x^{\prime}} \right)} \right) $$

The mean function m(x) can be specified by using the explicit basis functions. Usually, the calculations are simplified by considering m(x) to be zero because it can be challenging to identify a fixed m(x)^41,45^. Thus, we have:

4 $$ f\left( {x_{L \cdot i} } \right) \sim GP\left( {0 \cdot k\left( {x \cdot x^{\prime}} \right)} \right) $$

The distribution of y is achieved by the combination of Eqs. (1) and (4):

5 $$ y \sim N\left( {0 \cdot k\left( {x \cdot x^{\prime}} \right) + \sigma_{noise}^{2} I_{n} } \right) $$

Considering all the above-described parameters and noises, we have:

6 $$ \left[ {\begin{array}{*{20}c} {\overrightarrow {{f_{L} }} } \\ {\overrightarrow {{f_{T} }} } \\ \end{array} } \right] \sim N\left( {0 \cdot \left[ { \begin{array}{*{20}c} {k\left( {x_{L} \cdot x_{L} } \right)} & {k\left( {x_{L} \cdot x_{T} } \right)} \\ {k\left( {x_{T} \cdot x_{L} } \right)} & {k\left( {x_{T} \cdot x_{T} } \right)} \\ \end{array} } \right]} \right) $$

7 $$ \left[ {\begin{array}{*{20}c} {\overrightarrow {{\varepsilon_{L} }} } \\ {\overrightarrow {{\varepsilon_{T} }} } \\ \end{array} } \right] \sim N\left( {0 \cdot \left[ {\begin{array}{*{20}c} {\sigma_{noise}^{2} I_{n} } & 0 \\ 0 & {\sigma_{noise}^{2} I_{n} } \\ \end{array} } \right]} \right) $$

The summation of Eqs. (6) and (7) gives the following Gaussian expression:

8 $$ \left[ {\begin{array}{*{20}c} {\overrightarrow {{y_{L} }} } \\ {\overrightarrow {{y_{T} }} } \\ \end{array} } \right] \sim N\left( {0 \cdot \left[ {\begin{array}{*{20}c} {k\left( {x_{L} \cdot x_{L} } \right) + \sigma_{noise}^{2} I_{n} } & {k\left( {x_{L} \cdot x_{T} } \right)} \\ {k\left( {x_{T} \cdot x_{L} } \right)} & {k\left( {x_{T} \cdot x_{T} } \right) + \sigma_{noise}^{2} I_{n} } \\ \end{array} } \right]} \right) $$

Then, the distribution of the y_T_ can be derived through the conditioning rule of Gaussians, in which μ_T_ and Σ_T_ are the mean value and the covariance:

9 $$ \left( {y_{T} |y_{L} } \right)\sim N\left( {\mu_{T} \cdot {\Sigma }_{T} } \right) $$

10 $$ \mu_{T} = m\left( {\overrightarrow {{y_{T} }} } \right) = k\left( {x_{T} \cdot x_{L} } \right)\left( {k\left( {x_{L} \cdot x_{L} } \right) + \sigma_{noise}^{2} I_{n} } \right)^{ - 1} \overrightarrow {{y_{T} }} $$

11 $$ \begin{aligned} {\Sigma }_{T} & = k\left( {x_{T} \cdot x_{T} } \right) = k\left( {x_{T} \cdot x_{T} } \right) + \sigma_{noise}^{2} I_{n} \\ & \quad - k\left( {x_{T} \cdot x_{L} } \right)\left( {k\left( {x_{L} \cdot x_{L} } \right) + \sigma_{noise}^{2} I_{n} } \right)^{ - 1} k\left( {x_{L} \cdot x_{T} } \right) \\ \end{aligned} $$

e given independent variable and the training data set can obtain the outputs prediction of the test data. In training, choosing a powerful kernel function, which has an invertible and symmetric matrix, could significantly affect the estimation power of the established GPR model. To find the most appropriate kernel function for the current study, the learning method was conducted, through which four common and diverse kernel functions of Matern, Exponential, Squared exponential, and Rational quadratic are manipulated. These functions have the following forms:

- Matern kernel function:
  12 $$ k_{M} \left( {x \cdot x^{\prime}} \right) = \sigma^{2} \frac{{2^{1 - v} }}{{{\Gamma }\left( v \right)}}\left( {\sqrt {2v} \frac{{x - x^{\prime}}}{\ell }} \right)^{v} K_{v} \left( {\sqrt {2v} \frac{{x - x^{\prime}}}{\ell }} \right) $$
- Exponential kernel function:
  13 $$ k_{E} \left( {x \cdot x^{\prime}} \right) = \sigma^{2} exp\left( { - \frac{{x - x^{\prime}}}{\ell }} \right) $$
- Rational quadratic kernel function:
  14 $$ k_{RQ} \left( {x \cdot x^{\prime}} \right) = \sigma^{2} \left( {1 + \frac{{x - x^{\prime 2} }}{2a\ell }} \right)^{ - a} $$
- Squared Exponential kernel function:
  15 $$ k_{SE} \left( {x \cdot x^{\prime}} \right) = \sigma^{2} exp\left( { - \frac{{x - x^{^{\prime} 2} }}{{\ell^{2} }}} \right) $$

where ℓ, α > 0, σ, and σ^2^ are the length scale, scale-mixture, amplitude, and variance. Also, the K_v_ and *v* represent the modified Bessel function and a positive parameter, respectively, while the symbol Γ indicates the gamma function. The exponential and squared exponential kernel functions are two special cases in the Matern function, where if *v* = 0.5 or 1 Matern function becomes exponential or squared exponential function.

### Data collection

A total number of 506 experimental data of CO_2_ adsorption by various structured MOFs, including pores decorated with open metal sites Cu_3_(BTC)_2_ and (MOF-505), hexagonally packed cylindrical channels (MOF-74), interpenetration (IRMOF-11), square channels (MOF-2), Mg_2_(dobdc), Cu-BTTri, the extra-high porosity MOF-177, Be-BTB, IRMOF-1, amino- and alkyl-functionalized pores (IRMOFs-3 and-6), and Co(BDP), were collected from reported studies (see Table S1)^17,40^. The pressure (P, bar), the temperature (T, K), the pore volume (V_p_, cm^3^/g), and the surface area (S, m^2^/g) of the MOFs are the model input variables, while the CO_2_ uptake (xCO_2_ mmol/g) is the output of the model. In order to establish the most accurate model, arbitrarily, 20% of the total data was separated as the testing set, which was used to study the validity of the model. The rest (80%) of the total data was utilized as the training set to investigate the MOF*-*CO_2_ systems. Five statistical parameters (Eqs. 16–20), including R^2^ (difference between the experiments and the calculated values), mean-square error (MSE), the standard deviation (STD), root-mean-square error (RMSE), and mean relative error (MRE) were used to evaluate the precision of the model.

16 $$ R^{2} = 1 - \frac{{\mathop \sum \nolimits_{i = 1}^{n} \left[ {x_{i}^{predicted} - x_{i}^{experimental} } \right]^{2} }}{{\mathop \sum \nolimits_{i = 1}^{n} \left[ {x_{i}^{predicted} - x_{m} } \right]^{2} }} $$

17 $$ STD = \sqrt {\mathop \sum \limits_{i = 1}^{n} \frac{{\left( {x_{i}^{predicted} - x_{m} } \right)^{2} }}{n}} $$

18 $$ MSE = \frac{1}{n}\mathop \sum \limits_{i = 1}^{n} \left( {x_{i}^{predicted} - x_{i}^{experimental} } \right)^{2} $$

19 $$ RMSE = \sqrt {\frac{{\mathop \sum \nolimits_{i = 1}^{n} \left( {x_{i}^{predicted} - x_{i}^{experimental} } \right)^{2} }}{n}} $$

20 $$ MRE = \frac{1}{n}\mathop \sum \limits_{i = 1}^{n} \frac{{\left| {x_{i}^{predicted} - x_{i}^{experimental} } \right|}}{{x_{i}^{experimental} }} $$

### Estimation of the precision of the collected data

**Figure 1:**
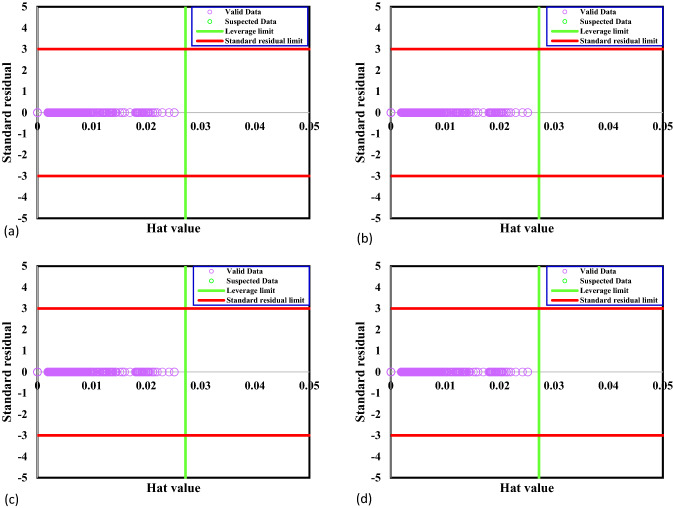
Detection of outliers for GPR model containing kernel function of (**a**) exponential, (**b**) matern, (**c**) squared exponential and (**d**) rational quadratic.

Some data have inconsistent behavior in the data bank with the remainder of the data points identified as the suspected data. The suspected data mainly makes mention of the experimental errors. Recognizing the suspected data is crucial because its presence in the data bank can result in an inappropriate forecast for the established model. Thus, to seek the suspected or outlier data and advance the data bank quality, the Leverage method is used. In this method, Hat matrix (H) and critical leverage limit (H*) are used for identification of the outlier data, which are defined as follow^46^.

21 $$ H = U\left( {U^{T} U} \right)^{ - 1} U^{T} $$

22 $$ H^{*} = \frac{3j}{{i + 1}} $$

where U, i, and j are a matrix dimensional of i * j, the number of the model parameters, and the number of training points, respectively. To investigate the precision of the CO_2_ adsorption data bank, the standardized residuals are represented against Hat values in Fig. 1, namely William's plot. The bounded zone between the critical leverage limit and standardized residuals of − 3 to 3 is known as the reliable region in William's plot. It is clear that all the extracted data points for the CO_2_ uptake by different MOFs are reliable. Therefore, the dataset is excellent for testing and training models.

## Results and discussion

### Analysis of sensitivity

**Figure 2:**
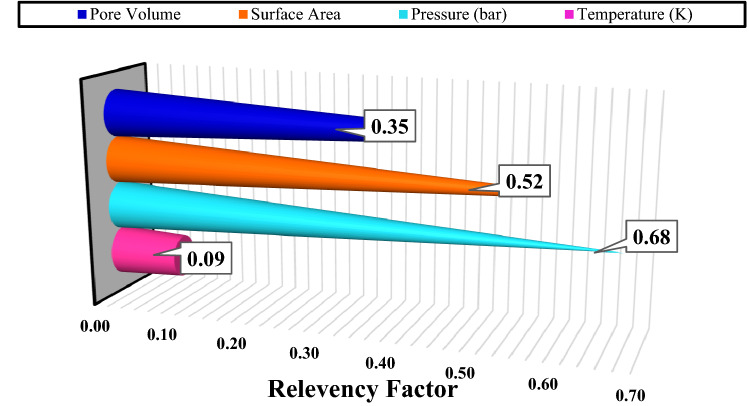
Analysis of sensitivity of the input parameters for CO_2_ uptake by various MOFs.

In order to propose a precise model, identification of the effects of the input on the CO_2_ uptake by MOFs is vital. A sensitivity analysis is the needed technique to obtain the relevancy factor of each input parameters, which is calculated as follow^47,48^:

23 $$ r = \frac{{\mathop \sum \nolimits_{i = 1}^{n} \left( {X_{k.i} - \overline{X}_{k} } \right)\left( {Y_{i} - \overline{Y}} \right)}}{{\sqrt {\mathop \sum \nolimits_{i = 1}^{n} \left( {X_{k.i} - \overline{X}_{k} } \right)^{2} \mathop \sum \nolimits_{i = 1}^{n} \left( {Y_{i} - \overline{Y}} \right)^{2} } }} $$

where ${X}_{k.i}$, ${\overline{X} }_{k}$, ${Y}_{i}$, and $\overline{Y }$ are the ‘k’ th input, input average, ‘i’th output, and the average of outputs, respectively. The more value of r for an input parameter means that its efficiency on the CO_2_ adsorption is higher and vice versa. The effect of the input variable on the CO_2_ adsorption is shown in Fig. 2. The sensitivity analysis indicates that the pressure and the surface area of MOFs with r values of 0.68 and 0.52 are the most influential input variables on the CO_2_ adsorption estimation. These inputs have a direct relationship with CO_2_ uptake. Furthermore, increasing the pore volume of the MOFs results in higher CO_2_ adsorption. It is worth mentioning that the small amount of r for the temperature can be related to its limited change in the experimental data.

### Modeling results

In order to examine how exactly the proposed model is, the matching statistical parameters are used to specify a match between experimental and predicted CO_2_ adsorption values. These parameters are determined and reported in Table 1. The R^2^ values of 1.00, 0.998, 0.997, and 0.997 are obtained for GPR models with Exponential, Matern, Squared exponential, and Rational quadratic kernel functions. The error parameters of MRE, MSE, RMSE, and STD in the training data indicate that the proposed GPR models have trained the data with acceptable precision. In addition to the prediction accuracy of the training data, the ability of the established models to forecast unseen CO_2_ adsorption data points has critical importance. Thus, the proposed models were assessed for the testing data set. It can be seen that the GPR model containing the Exponential kernel function has the most accurate prediction of the unseen CO_2_ uptake dataset, where R^2^, MRE, MSE, RMSE, and STD are 0.999, 3.11%, 0.07, 0.26, and 0.22, respectively.

**Table 1:** The statistical parameters of proposed GPR models.

	R^2^	MRE (%)	MSE	RMSE	STD
**GPR (Exponential)**					
Train	1.000	0.51	0.00	0.02	0.02
Test	0.999	3.11	0.07	0.26	0.22
Total	1.000	1.75	0.02	0.26	0.14
**GPR (Matern)**					
Train	0.998	3.06	0.14	0.38	0.35
Test	0.990	62.78	0.65	0.81	0.75
Total	0.995	31.13	0.28	0.81	0.49
**GPR (Squared exponential)**					
Train	0.997	1.68	0.20	0.44	0.41
Test	0.992	41.81	0.56	0.75	0.69
Total	0.995	20.40	0.33	0.75	0.53
**GPR (Rational quadratic)**					
Train	0.997	10.27	0.20	0.45	0.40
Test	0.989	36.59	0.65	0.81	0.72
Total	0.994	22.83	0.36	0.81	0.53

**Figure 3:**
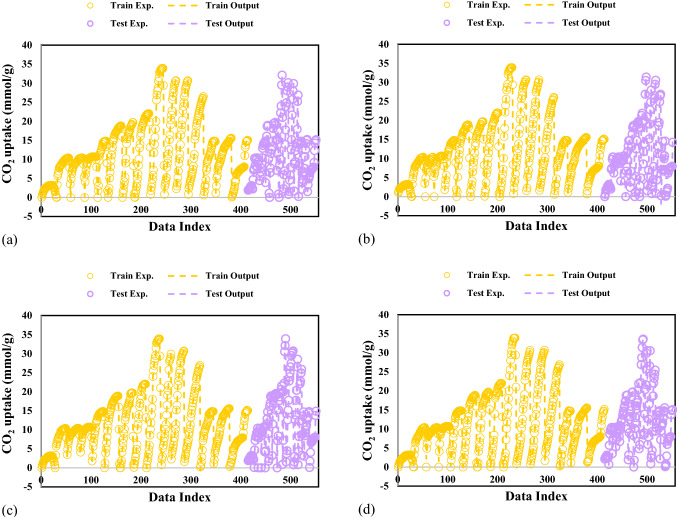
Comparison of experimental values and model outputs for GPR model containing kernel function of (**a**) Exponential, (**b**) Matern, (**c**) Squared exponential and (**d**) Rational quadratic.

To further confirm the precision of the established models, the experimental and predicted CO_2_ adsorption values are simultaneously shown in Fig. 3. It can be clearly observed that there is excellent agreement between the experimental CO_2_ adsorptions and different GPR models. For all proposed models, the predicted CO_2_ adsorption values follow the experimental CO_2_ adsorption precisely. Thus, the proposed GPR models have outstanding capability in the prediction of CO_2_ adsorption.

**Figure 4:**
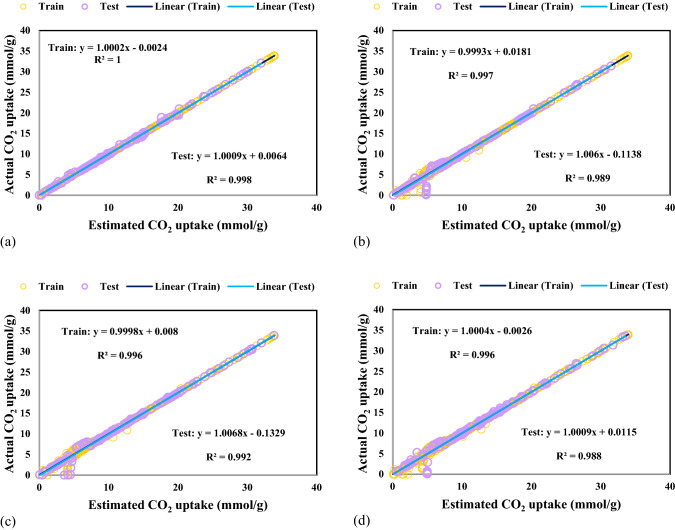
Cross plots for GPR model containing kernel function of (**a**) Exponential, (b**)** Matern, (**c**) Squared exponential and (**d**) Rational quadratic.

The predicted CO_2_ adsorption values versus experimental data for all the models are plotted and described in Fig. 4. All the predicted CO_2_ adsorption are situated to their experimental values so that the fitting lines on them have correlation coefficients higher than 0.98. The fitting lines cross considerably with 45° line representing the precision of all the GPR models for forecasting experimental CO_2_ adsorption data. The bisector line (45° line) is a standard for the precision of established models. Nevertheless, the GPR model with Exponential kernel function yields the most precise results due to the correlation coefficient of 1.

**Figure 5:**
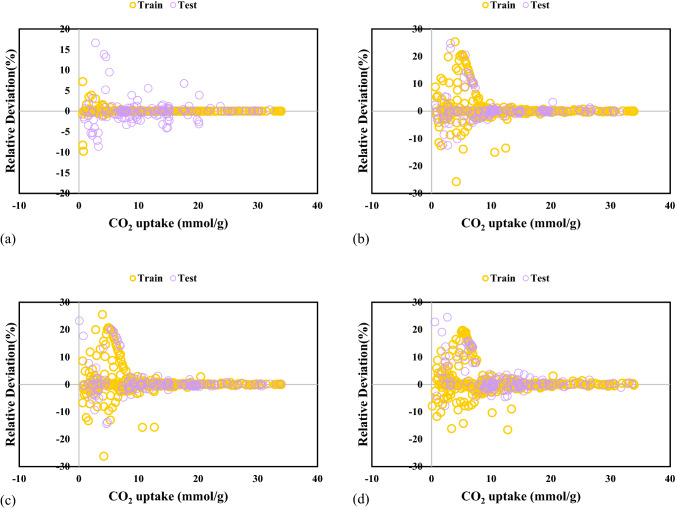
Comparison of experimental values and model outputs for GPR model containing kernel function of (**a**) Exponential, (**b**) Matern, (**c**) Squared exponential and (**d**) Rational quadratic.

Figure 5 shows the relative deviations between the experimental CO_2_ adsorption and all GPR models' predicted values. As it is presented, the various kernel functions of Matern, Squared exponential, and Rational quadratic have absolute deviation points lower than 30%, while for Exponential kernel function, they are lower than 20%.

According to the results, the proposed GPR models showed excellent performance for CO_2_ adsorption prediction. To ensure that the suggested models have enough precision in estimating CO_2_ adsorption by different MOFs, the current study results are compared to the available correlations with the same aim reported by Dashti et al.^25^. The statistical parameters, including R^2^, MSE, and STD, for the Dashti et al. study are listed in Table S2. Among the four examined algorithms, the RBF showed the best prediction with R^2^ = 0.997, MSE = 0.204, and STD = 4.211. In comparison, all the established GPR models have better estimating of CO_2_ adsorption, specifically, the GPR model with Exponential kernel function with R^2^ = 1.00, MSE = 0.02, and STD = 0.14.

**Figure 6:**
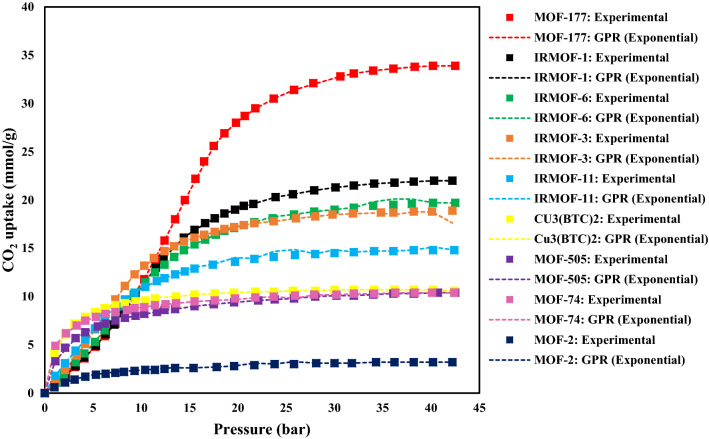
The CO_2_ adsorption capacities of different MOFs at 298 K.

As shown in Fig. 6, MOF-177 has the highest CO_2_ adsorption capacity of 33.5 mmol/g, which is much more significant than other MOFs. After that, IRMOFs-11, -1, and -3, with Zn_4_O(O_2_C)_6_-type frameworks, show excellent capacities for CO_2_ adsorption at room temperature. These MOFs have great effective pore sizes, which induce a sigmoidal shape(step) in their adsorption isotherms^24^. Also, the CO_2_ adsorption isotherms of MOF-2, MOF-74, Norit RB2, MOF-505, and Cu_3_(BTC)_2_ are monotonic (Type I). The severe CO_2_ adsorption at low pressure makes a “knee shape” in these isotherms, while the maximum capacity is gained at high pressure as the pores are saturated.

**Figure 7:**
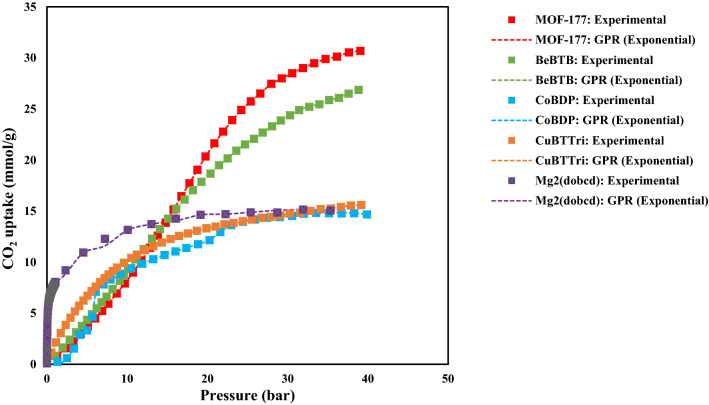
The CO_2_ adsorption capacities of different MOFs at 313 K.

**Figure 8:**
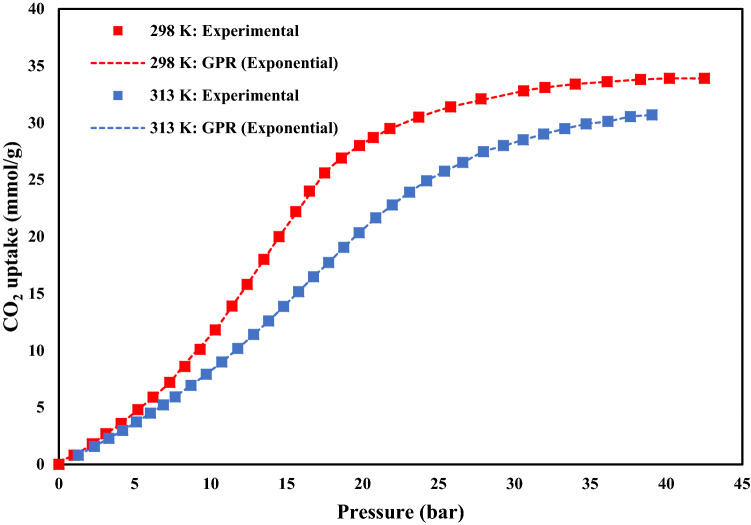
The CO_2_ adsorption capacities of MOF-177 at 298 and 313 K.

Figure 7 indicates the CO2 adsorption isotherms of Co(BDP), Cu-BTTri, BeBTB, Mg_2_(dobdc), and MOF-177 at 313 K. The MOF-177 and BeBTB show much better performance than other MOFs in the CO_2_ adsorption, which is due to their higher surface area (see Table S1). The isotherm of Co(BDP) has a step-like feature which might be attributed to its flexible structure, allowing gate-opening occurrence^49,50^. Cu-BTTri and Mg_2_(dobdc) adsorbed high CO_2_ at low pressures, which is related to their surface areas and the additional polarizing effect of metal cations on the framework surface. Due to higher polarizability and the quadrupole moment of CO_2_, the surface area can affect the amount of CO_2_ adsorption by MOF. Figure 8 shows the temperature effect on the CO_2_ adsorption.

## Conclusion

In the current study, the GPR models based on different kernel functions have been established to estimate the CO_2_ adsorption ability of MOFs in terms of pressure, temperature, pore volume, and surface area of MOFs. For this purpose, 506 experimental CO_2_ uptake values in the literature have been collected and assessed. Four various kernel functions of Exponential, Squared exponential, Matern, and Rational quadratic have been studied. An excellent match has been detected between the experimental CO_2_ adsorptions and predicted values by the developed GPR models, confirming these models' great ability in determining the CO_2_ uptake. Among the proposed models, the GPR model based on exponential kernel function, was shown as the most precise predictive tool with R^2^ = 1.00, MSE = 0.02, and STD = 0.14. Also, the suggested GPR models have better performance in comparison to the reported correlations. The sensitivity analysis indicates that the pressure is the most influential variable in CO_2_ adsorption by MOFs. The surface area of the MOFs can be presented as the second determining paramater in the CO_2_ capture by MOFs systems. The discussions in the current study can make it a helpful report for the engineers and researchers dealing with gas separation technologies.

## Data availability

The data that support the findings of this study are available from the corresponding author upon reasonable request.

## Supplementary Information

Supplementary Information.

## Abbreviations

ANFIS: Adaptive neuro fuzzy inference system
ANN: Artificial neural network
BDP: 1,4-Benzenedipyrazolate
Be-BTB: Beryllium benzene tribenzoate
BTC: Benzene-1,3,5-tricarboxylate
BTTri: 1,3,5-Benzenetristriazolate
GPR: Gaussian process regression
H: Hat matrix
H*: Critical leverage limit
I_n_: Unit array
k: Covariance (kernel) function
K: Modified Bessel function
ℓ: Length scale
LS-SVM: Least square-support vector machine
m: Mean function
MSE: Mean square error
MOF: Metal organic framework
MRE: Mean relative error
P: Pressure (bar)
T: Temperature (K)
r: Relevancy factor
R^2^: Difference between the experiments and the calculated values
RBF: Radial basis function
RF: Random forest
RMSE: Root mean square error
S: Surface area (m^2^/g)
STD: Standard deviation
U: Matrix dimensional of i * j
V: Volume (cm^3^/g)
x: Input variable
$X$: Input parameter in r eq.
${\overline{X} }$: Input average in r eq.
y: Target variable
$Y$: Output parameter in r eq.
$\overline{Y }$: Average of outputs in r eq

## Greek symbols

*f*: A random function
$$\varepsilon $$: Observation noise
σ^2^_noise_: Variance of the noise
μ_T_: Mean value
Σ_T_: Covariance
α: Scale mixture
σ: Amplitude
Γ: Gamma function

## Suscripts

*E*: Exponential kernel function
*i*: Number of parameters
*j*: Number of training points
*k*: Number of input parameter in r eq.
*M*: Matern kernel function
*p*: Pore
*RQ*: Rational quadratic kernel function
*SE*: Squared exponential kernel function
*T*: Testing data
*v*: Positive parameter
