# Supplementary material for: Towards estimation of CO2 adsorption on highly porous MOF-based adsorbents using gaussian process regression approach
Source: Sci Rep. 2021 Aug 3;11:15710. doi: 10.1038/s41598-021-95246-6 (PMC8333052; doi:10.1038/s41598-021-95246-6)
Supplement: Supplementary file 1 — Supplementary Information. [file 41598_2021_95246_MOESM1_ESM.docx]

**Towards Estimation of CO_2_ Adsorption on Highly Porous MOF-based Adsorbents Using Gaussian Process Regression Approach**

Majedeh Gheytanzadeh^1^, Alireza Baghban^2,*^ , Sajjad Habibzadeh^1,3,*^, Amin Esmaeili^4^, Otman Abida^5^, Ahmad Mohaddespour^5^ and Muhammad Tajammal Munir^5^

*^1^ Surface reaction and advanced energy materials laboratory, Chemical Engineering Department, Amirkabir University of Technology (Tehran Polytechnic), Tehran, Iran*

*^2^ Chemical Engineering Department, Amirkabir University of Technology (Tehran Polytechnic), Mahshahr Campus, Mahshahr, Iran*

*^3^Department of Chemical Engineering, McGill University, 3610 University Street, Montreal, QC H3A 0C5, Canada*

*^4^ Department of Chemical Engineering, School of Engineering Technology and Industrial Trades, College of the North Atlantic - Qatar, Doha, Qatar*

*^5^ College of Engineering and Technology, American University of the Middle East, Kuwait*

**Corresponding authors: Alireza_baghban@alumni.ut.ac.ir (A. Baghban); sajjad.habibzadeh@mail.mcgill.ca (S.Habibzadeh)*

**Table S1**. The details of the experimental data and the properties of the MOFs used in current study.

| No. | MOF | V_p_ (cm^3^/g) (Refs.) | Pressure range (bar) | Temperature range (K) | S (m^2^/g) | CO_2_ uptake (mmole/g) | No. Data | Refs. |
| --- | --- | --- | --- | --- | --- | --- | --- | --- |
| 1 | MOF-2 | 0.227 ^1^ | 0-42.2 | 298-298 | 345 | 0-3.2 | 39 | ^2^ |
| 2 | MOF-74 | 0.4 ^3^ | 0-42.4 | 298-298 | 816 | 0-10.4 | 31 | ^2^ |
| 3 | MOF-505 | 1.83 ^4^ | 0-42.5 | 298-298 | 1547 | 0-10.4 | 31 | ^2^ |
| 4 | Cu_3_(BTC)_2_ | 0.43 ^5^ | 0-42.4 | 298-298 | 1781 | 0-10.7 | 30 | ^2^ |
| 5 | IRMOF-11 | 0.92 ^6^ | 0-42.4 | 298-298 | 2096 | 0-14.8 | 32 | ^2^ |
| 6 | IRMOF-3 | 1.07 ^7^ | 0-42.2 | 298-298 | 2160 | 0-18.9 | 37 | ^2^ |
| 7 | IRMOF-6 | 1.14 ^7^ | 0-42.5 | 298-298 | 2516 | 0-19.7 | 37 | ^2^ |
| 8 | IRMOF-1 | 0.18 ^8^ | 0-42.2 | 298-298 | 2833 | 0-22.0 | 35 | ^2^ |
| 9 | MOF-177 | 1.59 ^9^ | 0-42.5 | 298-313 | 4508 | 0-33.9 | 69 | ^2,9^ |
| 10 | CuBTTri | 0.713 ^9^ | 0.5460-39.866 | 313-313 | 1750 | 1.16-16.99 | 43 | ^9^ |
| 11 | MG_2_(dobdc) | 0.573 ^9^ | 0.0005-35.317 | 313-313 | 1800 | 0.09-15.15 | 51 | ^9^ |
| 12 | CoBDP | 0.93 ^9^ | 1.3102-38.288 | 313-313 | 2030 | 0.28-16.56 | 31 | ^9^ |
| 13 | BeBTB | 1.701 ^9^ | 1.9600-38.834 | 313-313 | 4030 | 1.79-30.17 | 40 | ^9^ |

**Table S2.** The statistical parameters of previous correlations ^10^

|  | PSO-ANFIS | DE-ANFIS | RBF | LSSVM |
| --- | --- | --- | --- | --- |
| R^2^ | 0.958 | 0.930 | 0.997 | 0.997 |
| MSE | 2.571 | 4.362 | 0.204 | 0.167 |
| STD | 23.16 | 13.78 | 4.211 | 6.988 |

**References**

1. Tranchemontagne, D. J., Hunt, J. R. & Yaghi, O. M. Room temperature synthesis of metal-organic frameworks: MOF-5, MOF-74, MOF-177, MOF-199, and IRMOF-0. *Tetrahedron* **64**, 8553–8557 (2008).

2. Millward, A. R. & Yaghi, O. M. Metal-organic frameworks with exceptionally high capacity for storage of carbon dioxide at room temperature. *J. Am. Chem. Soc.* **127**, 17998–17999 (2005).

3. Srinivas, G., Krungleviciute, V., Guo, Z. X. & Yildirim, T. Exceptional CO2 capture in a hierarchically porous carbon with simultaneous high surface area and pore volume. *Energy Environ. Sci.* **7**, 335–342 (2014).

4. Zhang, M. *et al.* Fine Tuning of MOF-505 Analogues To Reduce Low-Pressure Methane Uptake and Enhance Methane Working Capacity. *Angew. Chemie - Int. Ed.* **56**, 11426–11430 (2017).

5. Senthil Kumar, R., Senthil Kumar, S. & Anbu Kulandainathan, M. Efficient electrosynthesis of highly active Cu3(BTC) 2-MOF and its catalytic application to chemical reduction. *Microporous Mesoporous Mater.* **168**, 57–64 (2013).

6. Yang, Q., Zhong, C. & Chen, J. F. Computational study of CO2 storage in metal-organic frameworks. *J. Phys. Chem. C* **112**, 1562–1569 (2008).

7. Rowsell, J. L. C. & Yaghi, O. M. Effects of functionalization, catenation, and variation of the metal oxide and organic linking units on the low-pressure hydrogen adsorption properties of metal-organic frameworks. *J. Am. Chem. Soc.* **128**, 1304–1315 (2006).

8. Ozkan, U. S. *Design of Heterogeneous Catalysts: New Approaches based on Synthesis, Characterization and Modeling*. *Design of Heterogeneous Catalysts: New Approaches based on Synthesis, Characterization and Modeling* (2009). doi:10.1002/9783527625321.

9. Herm, Z. R., Swisher, J. A., Smit, B., Krishna, R. & Long, J. R. Metal-organic frameworks as adsorbents for hydrogen purification and precombustion carbon dioxide capture. *J. Am. Chem. Soc.* **133**, 5664–5667 (2011).

10. Dashti, A., Bahrololoomi, A., Amirkhani, F. & Mohammadi, A. H. Estimation of CO2adsorption in high capacity metal-organic frameworks: Applications to greenhouse gas control. *J. CO2 Util.* **41**, (2020).
